# The Psychotic Impact of Helicobacter pylori Gastritis and Functional Dyspepsia on Depression: A Systematic Review

**DOI:** 10.7759/cureus.5956

**Published:** 2019-10-21

**Authors:** Asmaa M Al Quraan, Nitya Beriwal, Pema Sangay, Tashi Namgyal

**Affiliations:** 1 Research, California Institute of Behavioral Neurosciences and Psychology, Fairfield, USA; 2 Research, California Institute of Behavioral Neurosciences and Psychology, Fairfield, IND

**Keywords:** helicobacter pylori, infection, anxiety, depression, functional dyspepsia, gastritis

## Abstract

The clinical practice of adding antidepressant drugs to the therapy for the eradication of Helicobacter pylori (H. pylori) in addition to the standard drug regimen is not yet well established. This study aims to establish if there is an association between H. pylori gastritis and depression and to further analyze the therapeutic effect of antidepressants on symptomatic relief in gastritis. A systematic review was done using articles collected within the last seven years without regional or language localization obtained using PubMed, PubMed Central® (PMC), Google Scholar, and the Cochrane controlled trials. The search terms included Helicobacter pylori, depression, functional dyspepsia, and antidepressants. We selected three randomized controlled trials (RCTs), eight cross-sectional studies, four prospective studies, one cohort study, and two review articles. Trials that were prescribed antidepressants for clinical improvement of dyspepsia in patients with H. pylori gastritis that showed no improvement after eradication therapy standard regimen were included. In conclusion, patients who showed no improvement in functional dyspepsia after H. pylori eradication were seen to improve on antidepressant therapy. Further investigation and studies to analyze this correlation are recommended.

## Introduction and background

A 24-year-old female patient, on her third visit to our clinic, presented with complaints of epigastric pain and dyspepsia for more than a 10-month duration. The patient had undergone an upper-endoscopy and subsequent biopsy, which was revealed on pathological examination and found to be consistent with H. pylori chronic gastritis. She was on a triple therapy regimen with a multi-modal approach over the last ten months, which also included 14 days of protocol-based management, following which no improvement in her symptoms was seen. In her second upper-endoscopy, two weeks later, similar findings were observed. On re-evaluation of the patient, it was found that her symptoms had flared up after she experienced sociological changes, which were leaving her job and her recent divorce. She felt lonely and attempted suicide multiple times during the last four months. She was then referred to a psychiatrist who prescribed her antidepressants in adjunction with the H. pylori standard drug therapy regimen. After one month of treatment, the patient’s symptoms relieved, and her quality of life improved. After that, she was found to be symptom-free. This is a hypothetical clinical scenario. We expect this study to emphasize the relationship between Helicobacter pylori gastritis and depression. We also intend to analyze any possible role of antidepressants in the treatment of chronic H. pylori gastritis.

Overview of H. pylori gastritis

H. pylori is a spiral-shaped, microaerophilic, gram-negative bacteria, approximately 3.5 microns in length and 0.5 microns in width. Its mobility through viscous solutions is enhanced with the help of two to seven unipolar sheathed flagella. It is catalase, oxidase, and urease positive. Urease is vital for its survival and colonization, which is produced in abundance, making up more than five percent of the organism's total protein weight. The bacterial urease activity is clinically essential, in that it forms the basis for several invasive and non-invasive tests to diagnose infection [1-4].

H. pylori affect people of all ages, with an average of about 50% of the world's population being affected in their lifespan. It is highly prevalent in younger population groups and developing countries as compared to industrialized nations. The risk of acquiring H. pylori infection is related to socioeconomic status and living conditions early in life. Factors such as the density of housing, overcrowding, number of siblings, sharing a bed, and lack of running water have all been linked to a higher acquisition rate of H. pylori infection. The infection persists once acquired and may or may not produce gastroduodenal disease. The definite route by which the infection spreads remains controversial. Person-to-person transmission of H. pylori occurs mainly through either feco-oral or trans-oral exposure. Re-infection with H. pylori following successful bacterial eradication is unusual. Recurrence of infection most commonly represents the recrudescence of the original bacterial strain [1]. 

H. pylori infection is almost always accompanied by gastritis, an alternate diagnosis should be suspected in the absence of the same. H. pylori gastritis typically starts as diffuse antral gastritis, and then subsequently spreads to the gastric corpus if left untreated. The changes of chronic active gastritis may be associated with intestinal metaplasia or dysplasia. Proximal migration of the organisms leading to corpus gastritis may be facilitated by the chronic use of proton pump inhibitors (PPIs). Acute inflammation disappears rapidly with treatment; however, chronic inflammation, including lymphoid follicles, can persist for years. Immunohistochemistry tests might be necessary for the detection of H. pylori organisms in patients on antibiotic therapy, chronic PPI therapy, or with other hypochlorhydria states that are predisposed to gastric bacterial overgrowth [5-8].

The pathophysiology of H. pylori infection and its eventual clinical outcome is a result of the complex interaction between the host and the bacterium, which is influenced by the environment and modulated by mainly several unidentified factors. H. pylori attach to the tissue, to subsequently release enzymes and other microbial products that cause cellular damage. Multiple strains of H. pylori with functional differences co-exist that may relate to their virulence and extent of tissue damage produced in the host. On the other hand, many types of virulence factors might get released from similar H. pylori strains, making it unclear as to which factors might be of utmost importance in the pathogenesis of the disease. Although H. pylori are non-invasive organisms, they stimulate a robust inflammatory and immune response. Bacterial colonization, persistence, and virulence, which result in an innate and adaptive host immune response, are all important in the pathogenesis of H. pylori-related disease [9-12].

Multiple treatment regimens exist to treat H. pylori infection; however, the optimal therapeutic regimen has not yet been defined (Table 1). For patients failing one course of H. pylori treatment, it is recommended either an alternate regimen using a different combination of medications as triple therapy or, preferably, quadruples therapy. The further treatment approach depends on the initial treatment, in that clarithromycin and antibiotics used previously should be avoided if possible. If patients failing two attempts at treatment, compliance with medications should be reinforced. Culture with antibiotic sensitivity testing is beneficial to guide subsequent therapies. For rescue therapy, levofloxacin (250 mg), amoxicillin (1 g), and a PPI each given twice daily for two weeks are often used [13].

**Table 1:** Different treatment regimens for Helicobacter pylori gastritis treatment Different treatment regimens for Helicobacter pylori gastritis treatment [13]

Regimen	Drugs	Duration
Triple therapy	1-Proton pump inhibitor (PPI), 2-Amoxicillin (1 g twice daily) ( substitution of amoxicillin with metronidazole (500 mg twice daily) only in penicillin-allergic individuals, since metronidazole resistance is common which reduce the efficacy of treatment) and 3-Clarithromycin (500 mg twice daily)	For 10 days to two weeks
Quadruple therapy can be used as initial therapy, it is recommended in the retreatment	1-PPI twice daily, 2-Bismuth (525 mg four times daily), 3-Two antibiotics ( metronidazole 250 mg four times daily and tetracycline 500 mg four times daily) Or With a commercially available combination capsule containing bismuth subcitrate, metronidazole, and tetracycline four times daily	For 10 to 14 days.
Rescue therapy	Levofloxacin (250 mg), Amoxicillin (1 g), and a PPI each given twice daily	For two weeks

**Figure 1:**
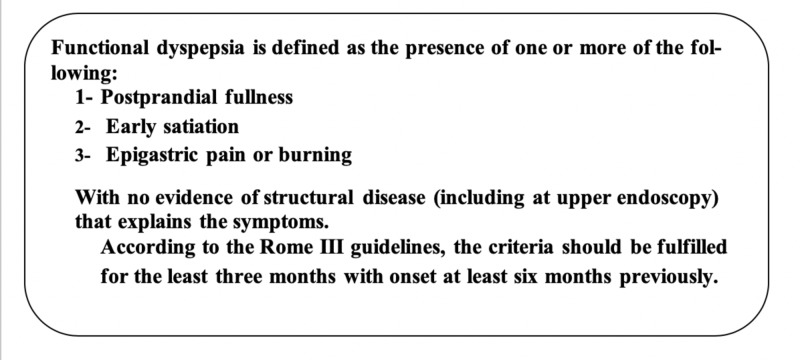
Rome III criteria of functional dyspepsia Rome III criteria of functional dyspepsia [15]

Functional dyspepsia is defined as the presence of one or more of the following: postprandial fullness, early satiety, and epigastric pain or burning with no evidence of structural disease (even on upper endoscopy) to explain the symptomatology of the patient. Patients with these symptoms and a negative diagnostic evaluation likely have functional dyspepsia, according to the Rome III guidelines (Figure 1) [14,15]. In 2016, Rome IV criteria defined that the diagnosis of functional dyspepsia required bothersome clinical symptoms, and the brain-gut axis was acknowledged as an important factor in the etiology of functional gastrointestinal disorders [16,17].

Cheung et al. concluded that there is up-regulation of transient receptor potential vanilloid (TRPV1 and TRPV2) and down-regulation of brain-derived neurotrophic factor (BDNF), despite lacking gastric mucosal inflammation as seen in patients with functional dyspepsia [18]. These conclusions suggest that there is a role in immune regulation in the pathogenesis of functional dyspepsia [18]. Tominaga et al. concluded in their study that the underlying pathogenesis of functional dyspepsia is the up-regulation of the serotonin transporter (SERT) levels in the midbrain and thalamus, which present as abdominal and psychological symptoms in patients as a result of brain-gut interaction [19]. We suggest that the refractory H. pylori infection is strongly related to depression, in that functional dyspepsia is a common symptom among patients having associated H. pylori infection. Since then, we suggest adding anti-depressant to the regimen along with antibiotic therapy in such cases.

## Review

The research data was collected to reveal the interconnection between depression and H. pylori gastritis, in particular, to identify if there is a synergistic effect on adding antidepressant drugs to the recommended standard regimen for H. pylori eradication. For a relevant, robust data search, a strict inclusion criterion was followed in the research. The studies which were published within the last seven years and only human studies were selected. It includes clinical trials, cross-sectional, prospective studies, and systematic review studies. All studies conducted globally were included, without regional or language localization. Keywords were used, and relevant studies were then selected from the search results based on their content and significance (Table 2).

**Table 2:** Data of relevant studies extracted on search using appropriate keywords

Keywords	Data collection
1-Helicobacter pylori gastritis and depression.	10
2-Depression and stomach.	12
3- Helicobacter pylori infection and serotonin.	0
4- Depression and dyspepsia.	19
5-Dyspepsia and serotonin.	0
6- Anxiety and stomach.	45
7-Anxiety and dyspepsia.	32
8-Helicobacter pylori and Tricyclic antidepressants (T.C.As).	0
9-Helicobacter pylori and Monoamine oxidase inhibitors(M.A.O.Is).	0
10- Stomach and brain axis	9

**Figure 2:**
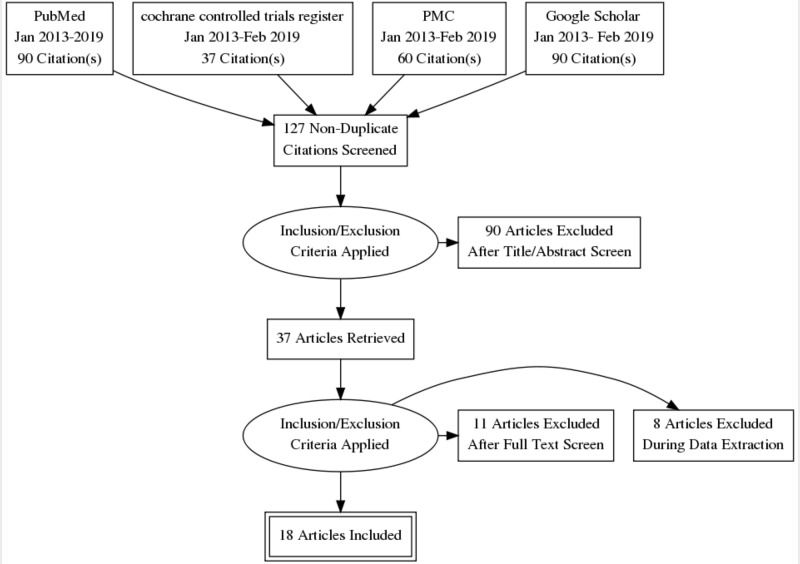
Data extraction and processing PMC: PubMed Central®

The keywords used for the search were: Helicobacter pylori (H. pylori) gastritis, depression, antidepressant drugs, stomach, brain, serotonin, dyspepsia, and anxiety. One hundred twenty-seven relevant studies were collected, of which 18 were selected after abstract-screening, removal of duplicates, and analysis of full paper contents (Figure 2).

All data was obtained lawfully while ensuring accurate reporting of the same. Necessary citation and confidentiality were also maintained. Data that was used strongly correlate the association between the trilogy H. pylori, depression and functional dyspepsia, and recommends the addition of antidepressant medications to the standard regimen therapy to treat H. pylori. Table 3 summaries some relevant data to emphasize the relation of H. pylori gastritis and depression.

**Table 3:** Summary of some relevant data in order to emphasize the relation of Helicobacter pylori gastritis and depression H. pylori: Helicobacter pylori, F.D: functional dyspepsia, PMC: PubMed Central®,  GI: gastrointestinal tract, HbA1c: Hemoglobin A1c, SSRIs: Selective Serotonin Reuptake Inhibitors

Study title	Study type	Published	Search method	Conclusion
Uninvestigated dyspepsia and associated factors of patients with gastrointestinal disorders in Dessie Referral Hospital, Northeast Ethiopia [20]	Cross-sectional study	2018	PMC, PubMed	H. pylori strongly associated with F.D
Endoscopic findings in uninvestigated dyspepsia [21]	Cross-sectional study	2014	Google Scholar	High prevalence of F. D with H. pylori
Functional dyspepsia and dyspepsia associated with Helicobacter pylori infection: Do they have different clinical characteristics? (Article in English, Spanish) [22]	Cross-sectional	Sept 2016	Google Scholar	High prevalence of F. D with H. pylori
Correlation between social factors and anxiety-depression in function dyspepsia: do relationships exist?[23]	Cross-sectional	Dec 2016	PMC	Depression strongly associated with F. D
Dyspeptic symptoms in patients with type 1 diabetes: endoscopic findings, Helicobacter pylori infection, and associations with metabolic control, mood disorders and nutritional factors [24].	Cross-sectional	Apr 2015	Google Scholar	Patients with diabetes mellitus type 1 who have dyspepsia are at great risk of having depression at high values of HbA1c and 34% have lesions similar to those having or not dyspepsia
Brain-based Correlations Between Psychological Factors and Functional Dyspepsia [25]	Cross-sectional study	Jan 2015	PMC	Depression strongly associated with F.D
Evidence that independent gut-to-brain and brain-to-gut pathways operate in the irritable bowel syndrome and functional dyspepsia: a 1-year population-based prospective study [26]	Prospective study	22 /July /2016	Google Scholar	Depression strongly associated F.D
Beliefs about GI medications and adherence to pharmacotherapy in functional GI disorder outpatients [27].	Cross-sectional		Google Scholar	People with F.D who are on antidepressant treatment show a great adherence to medication for their comorbidities than those not prescribed antidepressants
The impact of psychiatric and extraintestinal comorbidity on quality of life and bowel symptom burden in functional GI disorders [28].	Cohort study	29 July 2014	Google Scholar	A strong association between F.D and depression
The effect of psychotherapy in improving physical and psychiatric symptoms in patients with functional dyspepsia [29].	Clinical trial	Jan 2015	PMC, PubMed	Psychotherapy is effective in relieving gastric symptoms in patients with F.D
Psychopharmacological approach with the usage of selective serotonin reuptake inhibitors in functional dyspepsia [30].	Clinical trial	Nov 2014	PMC	Using antidepressant in adjunction with quadruple therapy reduces the prevalence of peptic ulcer among patient with anxiety and depression
Psychological effects of Helicobacter pylori-associated atrophic gastritis in patients under 50 years: A cross-sectional study [31].	Cross-sectional	Dec 2017	Google Scholar	H. pylori Seropositive patients show a high risk of having psychological distress and depression
Acute Neuropsychiatric Symptoms Associated with Antibiotic Treatment of Helicobacter pylori Infections: A Review [32].	Review	Jan 2017	Google Scholar	Neuropsychiatric symptoms may occur during the treatment of H. Pylori and rapidly resolute after discontinuation of treatment
Clinical observation on effect of flupentixol and melitracen combined with quadruple therapy on peptic ulcer patients with anxiety and depression (china) [33].	Clinical trial	2015	Google Scholar	Using SSRI in patients with F.D significantly reduce dyspeptic symptoms as well as anxiety and depression
Helicobacter pylori infection impacts on functional dyspepsia in Thailand [34].	Prospective study	2014	Google Scholar	F.D patients commonly have H. pylori and depression, and eradication of H. pylori is the key for treatment.
Ongoing symptoms after eradication of Helicobacter pylori: psychiatric disorders may accompany [35].	Prospective study	Jan 2013	PMC	Psychiatric illness should be excluded in patients who fail symptoms treatment after H. pylori eradication.
Prevalence of Helicobacter pylori Infection and Stress, Anxiety or Depression in Functional Dyspepsia and Outcome after Appropriate Intervention [36].	Prospective study	August 2017	PMC	Dyspepsia score in patients with F.D decreased dramatically after eradication therapy of H. pylori and psychiatric intervention in H. pylori-positive patients
A review of drug therapy for functional dyspepsia [37].	Review	August 2013	Google Scholar	Symptom relief of F.D after H. pylori eradication and the use of antidepressants in a small dose

**Figure 3:**
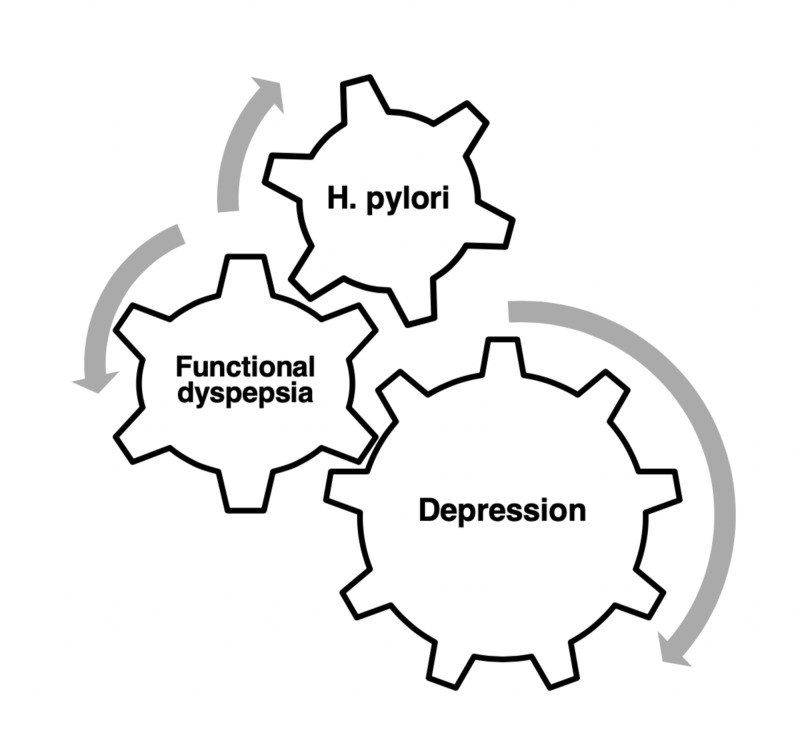
The inter-relationships between depression, H. pylori infection, and functional dyspepsia

Do antidepressant drugs alongside antibiotics play a role in the eradication therapy for H. pylori gastritis? What begins first? Is it the H. pylori gastritis or depression in patients suffering from chronic gastritis? One study showed that people who were negative for H. pylori, after exposure to stressful conditions, became positive for H. pylori infection. The explanation behind it was that people under stressful conditions develop low immunity, so they are more liable for contracting the infection [38]. The relationship between depression and H. pylori gastritis is emphasized in this study with the help of reviewing functional dyspepsia in between, and if antidepressant drugs play any role in treating patients with chronic H. pylori gastritis. Overall, the evidence is not sufficiently strong to determine the effectiveness of using antidepressants as an adjuvant therapy to the standard regimen (triplet or quadruplet drug therapy) in the treatment of H. pylori gastritis (Table 1). One of the symptoms of H. pylori gastritis is functional dyspepsia, which is characterized according to Rome III committee definition as the presence of one or more dyspepsia symptoms that originates from the gastroduodenal region, in the absence of any organic, systemic, or metabolic disease that is likely to explain the symptoms [39]. Functional dyspepsia symptoms include postprandial fullness, early satiety, postprandial nausea, excessive belching, epigastric pain, epigastric burning, and upper abdominal bloating [40]. To prove a strong relationship between H. pylori gastritis and depression, we review and discuss the following associations: between H. pylori gastritis and functional dyspepsia, functional dyspepsia and depression, Helicobacter pylori gastritis and depression, and finally, associations between the trilogy Helicobacter pylori, depression and functional dyspepsia (Figure 3).

Helicobacter pylori gastritis and functional dyspepsia

A cross-sectional study by Seid et al. studied 318 patients over the period September 1 - December 30, 2015, in Ethiopia and concluded that H. pylori infection should be ruled out in patients with uninvestigated dyspepsia and recommended starting eradication therapy [20]. The enrolled sample answered a questionnaire for collecting data regarding sociodemographic, lifestyle, and functional gastrointestinal disorders, and the diagnosis of dyspepsia was made according to the Rome III criteria. H. pylori infection was assessed using the stool antigen test [20]. Faintuch et al. enrolled 306 subjects in their cross-sectional study with uninvestigated dyspepsia, of which 282 patients answered a questionnaire according to Rome III criteria and underwent esophagogastroduodenoscopy [21]. The rapid urease test was applied to fragments of the antral mucosa, and epidemiological data were collected from the studied population. The mean age of patients was 44 years, in which 65% of the sample was women. They found: 45% of the patients reported alarm symptoms (unplanned significant weight loss over a short period of time), although cancer was not found, 66% of the patients were found to have functional dyspepsia, 18% had gastroesophageal reflux, 13% had ulcers, and 54% of them were found to have H. pylori infection. Their study resulted in finding a high prevalence of patients with dyspepsia and H. pylori infection [21]. In another cross-sectional study, Rodríguez-García et al. enrolled 578 patients with dyspepsia with no endoscopic lesions and divided them into two groups according to having (n=336) or not having H. pylori infection (n=242) [22]. Then, they were compared according to clinical characteristics, medical history, comorbidities, and usage of health resources. They found 58% of cases with dyspeptic symptoms have H. pylori infection without detectable endoscopic lesions [22].

Functional dyspepsia and depression 

Huang et al. enrolled 907 patients with functional dyspepsia that fulfilled the Rome III criteria and validated them using the Chinese version of the self-rating scale and self-rating anxiety scale [23]. Five hundred and sixteen of the sample have functional dyspepsia and anxiety-depression status. They found that patients with functional dyspepsia have a higher rate of anxiety and depression, commonly in female patients, advanced age, high-stress occupation, lower education level, and poor health condition [23]. Faria et al. found that people with type 1 diabetes mellitus (insulin-dependent) who have dyspepsia are at a greater risk of having depression at higher values of hemoglobinA1c (HbA1c) and low values of body mass index (BMI) [24]. All patients in the study were screened by endoscopy to look for lesions. Thirty-four percent of them were found to have lesions with similar frequency in patients having or not having dyspepsia. Concluding, there was no association between the symptoms and having an infection [24]. Nan et al. suggested that the altered cerebral glycometabolism may be a consequence of the vicious cycle of psychological vulnerability and gastrointestinal symptoms [25]. Patients with functional dyspepsia after controlling dyspepsia had increased glucose metabolism in the insula, anterior cerebral cortex, middle cingulate cortex, and middle frontal cortex related to depression score. Even patients with functional dyspepsia without anxiety or depression have an increase in glucose metabolism in the regions mentioned, and it is a 4-fold increase in metabolism as compared to normal healthy subjects. The methods of their study were based on fluorine-18-deoxyglucose positron emission tomography-computed-tomography. The altered glycometabolism was investigated in 40 patients with functional dyspepsia in comparison to 20 controls during resting state using statistical parametric mapping software [25].

Koloski et al. explained the independent gut-to-brain and brain-to-gut pathways that operate in the irritable bowel syndrome and functional dyspepsia [26]. It was a one-year population-based prospective study to determine which begins first in the pathogenesis of functional gastrointestinal disorders-the gut or the brain and their correlation with each other. They enrolled in 1900 people who completed a one-year follow-up survey subsequently. The survey contained questions based on Rome III criteria for functional dyspepsia and irritable bowel syndrome, besides hospital anxiety and depression scale. The result was that higher levels of anxiety and depression at baseline were a significant predictor of developing functional dyspepsia and irritable bowel syndromes over a one year follow up in these patients. Patients at the baseline who had functional dyspepsia and irritable bowel syndrome reported significantly higher levels of depression and anxiety on the one-year follow-up. One-third were mood disorders preceding the functional dyspepsia disorder, while, in two-third, the functional dyspepsia preceded mood disorder. In conclusion, in a major subset, the gut symptoms begin first, followed by the development of psychological distress [26].

In a prospective study of 536 patients with the mean age of 54.7 + 0.7 years, patients were enrolled over a 5.5 years interval and completed the beliefs about medications questionnaire (BMQ); 341 were with functional gastrointestinal dyspepsia (FGID) (irritable bowel syndrome 64.8%, functional dyspepsia 51.0%) and 142 were with structural gastrointestinal disease (SGID) (26.5%) (inflammatory bowel disease 28.9%, while gastroesophageal reflux 23.2%) [27]. Proton pump inhibitors (n=231), tricyclic antidepressants (n=167), and anxiolytics (n=122) were common prescribed medications. The FGID and SGID were similar across all the BMQ domains (P>0.05 for overuse, harm, necessity, and concern). The FGID had less necessity-concern framework (NCF) scores compared with the SGID subjects. The medication adherence in the FGID was correlated negatively with concern about medication harm (r=-0.24, P<0.001) and overuse (r=-0.15, P=0.001); however, higher N.C.F differences predicted medication compliance. The medication concern and overuse scores were correlated with psychiatric comorbidity among FGID patients (p<0.03 for each). Patients with FGID prescribed tricyclic antidepressants (TCA) (n=142) expressed a greater medication necessity (p=0.024) and found their gastrointestinal regimen to be more helpful (p=0.054), whereas patients with FGID not prescribed TCAs expressed a greater apprehension about medication overuse (p=0.002) on the BMQ. They concluded that subjects with FGID reported medication necessity and concern scores comparable to subjects with SGID but have negative perceptions about medications, particularly in the presence of psychiatric comorbidity; these factors may affect the treatment adherence and willingness to initiate neuromodulator regimen [27]. A cohort study enrolled 912 outpatients in gastrointestinal diseases clinic run by Vu et al. with mean age 47.2 + 1.5 years and consisting of 75.8% females. Six hundred and six of these patients (66.4%) met the Rome III irritable bowel syndrome or functional dyspepsia criteria or both [28]. Anxiety, depression, and extraintestinal functional dyspepsia comorbidities are common among patients with functional gastrointestinal disorder and showed a decrease in the health-related quality of life [28]. Faramarzi et al. emphasized in their clinical trial study that brief core conflictual relationship theme (CCRT) psychoanalytic psychotherapy can serve as an effective intervention for promoting relief in gastrointestinal and psychiatric symptoms in patients with functional dyspepsia [29]. Korendovych et al. proved that using selective serotonin reuptake inhibitor (SSRI) escitalopram in patients with functional dyspepsia (FD) reduces the dyspeptic symptoms as well as a significant decrease in anxiety and depression along with improvement of quality of life [30].

Helicobacter pylori gastritis and depression

Takeoka et al. concluded that patients with atrophic gastritis, regardless of H. pylori infection status, are significantly at a higher risk of experiencing psychological distress [31]. Females below 50 years of age with seropositive H. pylori gastritis showed the highest risk of psychological distress and depression using H. pylori seronegative status as the reference [31]. Neufeld et al. recommended physicians to remain alert regarding the possibility of occurring neuropsychiatric symptoms during the treatment of H. pylori infection and found a rapid resolution of symptoms typically after the discontinuation of the antibiotics [32]. Wang et al. proved that using flupentixol and melitracen combined with quadruple therapy could significantly improve the therapeutic efficacy and effectively reduce the recurrence rate in peptic ulcer patients with anxiety and depression [33].

Helicobacter pylori, depression and functional dyspepsia

Piriyapong et al. found that patients with functional dyspepsia commonly have H. pylori infection, anxiety, and depression and that it is more prevalent in postprandial dyspepsia than epigastric pain dyspepsia [34]. They concluded that H. pylori eradication might be the key to success for the treatment of F.D patients and to prevent the development of gastric cancer [34]. Ünal et al. enrolled 54 patients with functional dyspepsia and H. pylori infection without knowing their psychiatric illnesses [35]. They found 22 of them to have at least one psychiatric illness; the most common was depression in about 13 patients. They concluded that psychiatric illness should be considered for patients who fail the treatment of symptoms after H. pylori eradication, as it will affect their treatment [35]. A prospective study by Kabeer et al. to determine the prevalence of H. pylori and depression in patients with functional dyspepsia and to assess the outcome in three months after appropriate intervention enrolled 120 patients with functional dyspepsia, who underwent upper endoscopy to confirm H. pylori infection with either of two tests- the urease test or histopathology [36]. Adding to that, patients had a patient health questionnaire-9 scale (PHQ-9) to assess depression. Then, they were divided into four groups: group A (positive(+ve) for H. pylori and depression, n=35) treated with H. pylori eradication and psychiatric intervention; group B (+ve for H. pylori and negative(-ve) for depression, n=31) treated with H. pylori eradication alone; group c (-ve for H. pylori and +ve for depression, n=33) treated with psychiatric intervention alone; and group D (-ve for H. pylori and depression, n=21) treated with proton pump inhibitor PPI. They found that 55% had H. pylori, 56% had depression, and 29% had both depression and H. pylori infection. Females exceeded males in psychiatric comorbidity with a ratio of 3:1 proportion. The dyspepsia score declined dramatically after H. pylori eradication therapy and psychiatric intervention, in that appropriate intervention is beneficial except for those patients with negative H. pylori and positive for depression group, which might need further investigation and drug intervention for stress, anxiety and/or depression [36]. Chen, in his review, concluded that H. pylori eradication in Asian patients could lead to a higher proportion of functional dyspepsia with symptom relief, unlike in Europe and the USA [37]. Using anxiolytic drugs and antidepressants are reported to have peculiar effects on functional dyspepsia, especially in refractory functional dyspepsia, in which tricyclic antidepressants and selective serotonin reuptake inhibitors at small doses are most often recommended [37].

## Conclusions

Our aim in this paper was to establish the relationship between depression and H. pylori gastritis. With the help of the studies reviewed above, we found that H. pylori play a role in immunity besides in the brain-gut axis, which links it with psychiatric disorders like depression. Furthermore, receptors for serotonin were up-regulated when patients had H. pylori gastritis. Functional dyspepsia is a common presenting symptom for H. pylori gastritis. Some patients with refractory functional dyspepsia respond well when antidepressants such as TCAs or SSRIs are added to the standard regimens against H. pylori. Further studies are recommended for the benefit of proper clinical application of the same and development of comprehensive drug regimens.
